# Methylenetetrahydrofolate Reductase C677T Polymorphism and Type 2 Diabetes Mellitus in Chinese Population: A Meta-Analysis of 29 Case-Control Studies

**DOI:** 10.1371/journal.pone.0102443

**Published:** 2014-07-21

**Authors:** Bo Zhu, Xiaomei Wu, Xueyuan Zhi, Lei Liu, Quanmei Zheng, Guifan Sun

**Affiliations:** 1 Department of Occupational and Environmental Health, School of Public Health, China Medical University, Shenyang, People's Republic of China; 2 Department of Clinical Epidemiology and Evidence Medicine, The First Hospital of China Medical University, Shenyang, People's Republic of China; 3 Liaoning Academy of Safety Science, Shenyang, People's Republic of China; 4 Key Laboratory of Endocrine diseases in Liaoning Province, The First Hospital of China Medical University, Shenyang, People's Republic of China; Nanjing Medical University, China

## Abstract

**Background:**

Methylenetetrahydrofolate reductase (MTHFR), a key enzyme in folate metabolism, had significant effects on the homocysteine levels. The common functional MTHFR C677T polymorphism had been extensively researched. Several studies had evaluated the relationship between MTHFR C677T polymorphism and type 2 diabetes mellitus (T2DM), but the results were still controversial in the Chinese Han population. This meta-analysis was conducted to evaluate the relationship between MTHFR C677T polymorphism and T2DM in the Chinese Han population.

**Methods:**

We searched the relevant studies in multiple electronic databases, which published up to December 2013. We reviewed and extracted data from all the included studies on the relationship between MTHFR C677T polymorphism and T2DM in the Chinese Han population. The odds ratios (ORs) and their 95% confidence intervals (95%CIs) were used to evaluate the relationship. Fixed-effects and random-effects meta-analysis were used to pool ORs by the heterogeneity. Publication bias and sensitivity analysis were also examined.

**Results:**

29 studies were finally included in our meta-analysis, which contained 4656 individuals with T2DM and 2127 healthy controls. There was a significant relationship between MTHFR C677T polymorphism and T2DM under dominant (OR: 1.70, 95% CI: 1.42–2.02), recessive (OR: 1.48, 95% CI: 1.21–1.80), homozygous (OR: 1.89, 95% CI: 1.47–2.42), heterozygous (OR: 1.58, 95% CI: 1.33–1.87), and additive (OR: 1.46, 95% CI: 1.28–1.68) genetic model in a random-effects model. Subgroup analysis also reached similar results. Sensitivity analysis indicated that the overall result were dependable.

**Conclusions:**

There was a significant relationship between MTHFR C677T polymorphism and T2DM in the Chinese Han population. The results of our meta-analysis suggested that MTHFR 677T allele might be a risk genetic factor of T2DM in the Chinese Han population.

## Introduction

Type 2 diabetes mellitus (T2DM) is one of public health problems, seriously affects individual life quality, and increases individual economic burden. WHO estimates the number of people with diabetes will increase by 114% between 2000 and 2030, and China will become the major site of diabetes epidemic. In a systematic review of 22 studies on diabetes prevalence in China from 2000 to 2010, it increased from 2.6% to 9.7% during this decade [1]. It is estimated that China will have 380 million patients with T2DM by 2025 [2]. However, the pathogenesis of T2DM remains unclear [3]. Currently, the research on genetic polymorphisms is one of the most attention areas in the pathogenesis of T2DM, and some studies indicate that genetic polymorphisms have critical roles in the etiology of T2DM [4], [5].

Methylenetetrahydrofolate reductase (MTHFR) is a critical enzyme involved in folate metabolism, which converts 5, 10- methylene tetrahydrofolate to 5-methyl tetrahydrofolate. Mice deficient in MTHFR have reduced S-adenosylmethionine and increased S-adenosylhomocysteine, show hyperhomocysteinemia and global DNA hypomethylation [6]. The MTHFR C677T polymorphism is the most important genetic variation, which causes hyperhomocysteinemia [7]. The C677T polymorphism is a C to T transition at base pair 677, which will lead to the amino acid transition from Ala to Val and is associated with reduction of MTHFR activity. The variation of MTHFR C677T polymorphism may decrease enzyme activity by 65% and increase plasma total homocysteine levels particularly in the conditions of low dietary folate [8]. Some studies suggested that elevated plasma total homocysteine was associated with insulin resistance, which was the major cause of T2DM [3], [9], [10]. Homocysteine exposure can decline the viability of insulin-secreting cells, reduce glucokinase phosphorylating ability, and diminish insulin secretory responsiveness, lead to cell death [11]. Therefore, the MTHFR C677T polymorphism has been widely considered a genetic candidate for T2DM [12].

In recent years, numerous studies had demonstrated an association between MTHFR C677T polymorphism and T2DM. However, the results were not consistent [13]–[19]. A systematic review on Arab ethnicity found that MTHFR C677T polymorphism was significantly associated with T2DM [14], but another systematic review found that there was no association between MTHFR C677T polymorphism and T2DM around the world, similar results were repeated for ethnic group (Asian, Caucasian, African) [13]. Furthermore, previous studies also showed that the prevalence of MTHFR C677T polymorphism varies in different geographical regions and ethnic groups [20], and people from different ethnic groups had different genetic susceptibility with T2DM[21]. These findings suggested the study on the association between MTHFR C677T polymorphism and T2DM should be based on one single ethnical population to provide a precise estimation. Therefore, we conducted a meta-analysis to evaluate the association between MTHFR C677T polymorphism and T2DM specifically in Chinese Han population.

## Materials and Methods

### Search Strategy and Identification of Relevant Studies

A search strategy was carried out in multiple electronic databases (Cochrane, EMBASE, PubMed, CQVIP, CNKI (China National Knowledge Infrastructure), CBM (China Biological Medicine Database), and Wanfang databases) before December 2013. The following subject terms were used for searching by ‘methylenetetrahydrofolate reductase or MTHFR’, ‘gene or polymorphism or genetic polymorphism’, ‘Chinese or China’, and ‘diabetes or mellitus or diabetes mellitus or T2DM’. The papers were limited on humans and published in English or Chinese. In order to further identify any additional relevant data, we carefully searched the references in the selected studies.

### Data Extraction

The data from all included studies were independently extracted by two authors (BZ and XW) according to a standard protocol. The third author (LL) resolved the disagreement between two authors. We excluded the studies that did not follow the inclusion criteria, that lacked of sufficient data, or that considered duplicated articles. If we found the same data in different studies, we used the data only one time. The following items were extracted from all included studies: the first author's name, year of publication, region (province), total number of study, gender, genotypic distribution, allele frequencies.

### Inclusion Criteria

We set the inclusion criteria according to the Strengthening the Reporting of Observational Studies in Epidemiology (STROBE) Statement[22]. a) Give information on the criteria and methods for selection. b) Describe laboratory methods, including source and storage of DNA, genotyping methods and platforms. c) Clearly define genetic variants using a widely used nomenclature system. d) State whether Hardy-Weinberg equilibrium was considered and, if so, how. e) Report numbers in each genotype category.

### Statistical Analysis

STATA 11.0 software (StataCorp, College Station, TX, USA) was used to perform the meta-analysis. We used five genetic models, which included dominant (TT+CT vs. CC), recessive (TT vs. CC+CT), homozygous (TT vs. CC), heterozygous (CT vs. CC), and additive (T vs. C) models. The odds ratios (ORs) and their 95% confidence intervals (95%CIs) were used to evaluate the association between MTHFR C677T polymorphism and T2DM. We used Chi-square-based Q-tests to assess the heterogeneity between the individual studies [23]. If there was a significant heterogeneity among the individual studies, the random-effect model (DerSimonian and Laird method) was carried out to assess the pooled OR. Otherwise, the fixed-effect model (the Mantel–Haenszel method) was carried out.

We also conducted meta-regression and subgroup analysis to explore the sources of heterogeneity. To assess the reliability of the outcomes in the meta-analysis, a sensitivity analysis was performed by excluding one study at a time. Publication bias was assessed using the Egger's test [24]. We also conducted the Duval and Tweedie nonparametric “trim and fill” procedure to further assess the effect of publication bias in each genetic model [25]. Hardy-Weinbery equilibrium (HWE) in controls was assessed by the goodness-of-fit x^2^ test in each included study. The significance set at the P<0.05 in all analyses.

## Results

### Characteristics of Including Studies

**Figure 1:**
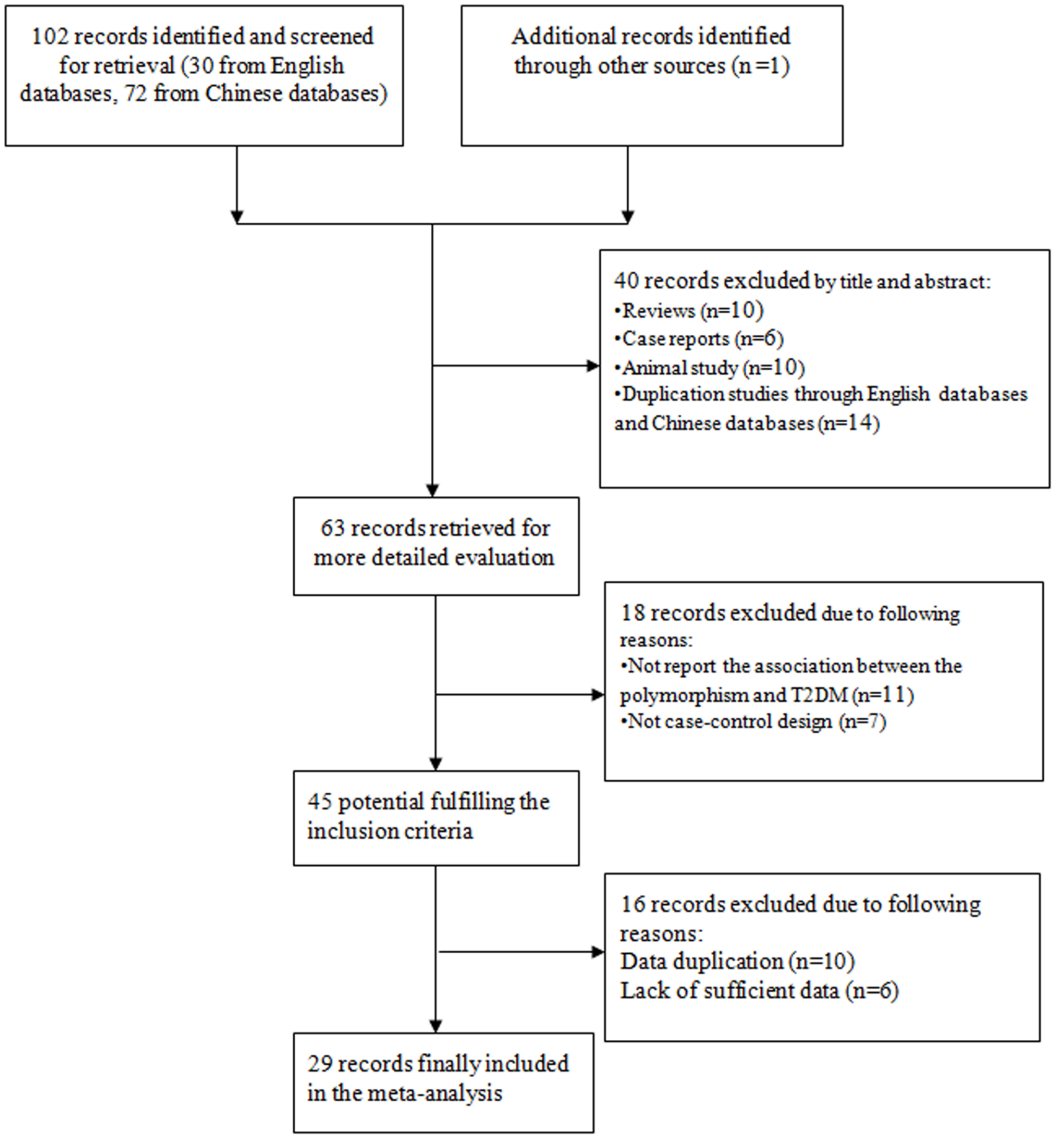
Flow diagram of included and excluded studies.

Figure 1 showed the procedure by which article was selected. A comprehensive search yielded 103 articles. After the removal of duplicated literatures and articles containing unspecific data that did not meet our criteria, a total of 29 studies was finally identified in our meta-analysis. Table 1 illustrated the characteristics of all the included studies in this meta-analysis. The data contained 4656 T2DM cases and 2127 healthy controls [15]–[17], [26]–[51]. The provinces of 29 studies included Heilongjiang, Beijing, Gansu, Shanxi, Zhejiang, Shanghai, Neimenggu, Guizhou, Tianjin, Guangdong, Hubei, Shandong, Jiangsu, Hebei, and Jilin. Except for 7 studies, the distribution of genotypes in the controls was consistent with HWE.

**Table 1:** Main characteristics of the 29 studies for meta-analysis.

Number	Author	Year	Region	Total number of study	Male (%)	Genotypic distribution						Allele frequencies				HWE
						CC		CT		TT		C		T		
						case	control	case	control	case	control	case	control	case	control	
1	Sun, Liang^a^	2013	Beijing	549	51.37	180	30	243	42	48	6	603	102	339	54	Yes
2	Mei, Qingbu	2012	Heilongjiang	215	No	17	17	51	70	23	37	85	104	97	144	Yes
3	Dai, Hongshuang^a^	2012	Heilongjiang	180	55.00	51	31	54	27	15	2	156	89	84	31	Yes
4	Chen, Airong	2010	Gansu	219	59.62	57	34	74	17	33	4	188	85	128	25	Yes
5	Zhang, Qiaohui^a,b,c^	2009	Shanxi	278	60.79	66	26	94	17	66	9	226	69	226	35	Yes
6	Qiu, Yi^a,b^	2009	Zhejiang	299	54.85	83	53	68	29	48	18	234	135	164	65	No
7	Hu, Ling^a,b^	2009	Shanxi	211	62.56	47	26	63	17	49	9	157	69	163	35	Yes
8	Wen, Jie	2008	Shanghai	211	52.13	43	27	82	25	29	5	168	79	140	35	Yes
9	Luo, Dan^a,b^	2008	Beijing	226	47.79	59	43	63	31	19	11	181	117	101	53	Yes
10	Chen, Ping	2008	Heilongjiang	240	No	19	14	70	73	27	37	108	101	124	147	No
11	Zhang, Chunyu^a,b,c^	2007	Neimenggu	141	51.77	28	34	29	19	19	12	85	87	67	43	No
12	Luo, Dan^a,b^	2007	Beijing	274	52.64	55	42	102	35	26	14	222	119	154	63	Yes
13	Yue, Hong^a,b,c^	2006	Shanxi	282	57.09	66	17	131	11	55	2	263	45	241	15	Yes
14	Xiao, Yan^a,b^	2006	Guizhou	146	No	16	47	53	25	4	1	85	119	61	27	Yes
15	Sun, Ying^a,b,c^	2006	Tianjin	355	60	113	47	85	25	68	17	311	119	221	49	No
16	Shi, Chengjun	2006	Guangdong	295	No	108	68	60	34	18	7	276	170	96	48	Yes
17	Liang, Wenchang	2005	Zhejiang	122	No	33	17	34	18	15	5	100	52	64	28	Yes
18	Guo, Lixin^a,b^	2005	Beijing	288	57.29	60	58	51	34	50	35	171	150	151	104	No
19	Sun, Jiazhong.	2005	Hubei	342	67.25	101	63	78	31	49	20	280	57	176	71	No
20	Zhou, Jun^a,b,c^	2004	Heilongjiang	208	No	16	8	78	31	45	30	110	47	168	91	Yes
21	Sun, Lei^a,b,c^	2004	Shandong	155	47.44	27	29	52	18	27	2	106	76	106	24	Yes
22	Mao, Li^a,b,c^	2004	Jiangsu	122	46.92	35	18	37	18	11	3	107	70	59	24	Yes
23	Chen, Airong^a,b,c^	2004	Gansu	126	64.29	24	21	45	9	22	5	93	51	89	19	No
24	Xu, Jinsheng^a,b,c^	2003	Hebei	175	45.14	30	7	54	25	39	20	114	39	132	65	Yes
25	Zhang, Guodong	2002	Shanghai	298	No	56	40	108	49	34	11	220	129	176	71	Yes
26	Shi, Jieping	2002	Jilin	106	No	12	22	31	29	7	5	55	55	45	45	Yes
27	Yang, Guoqing^a,c^	2001	Beijing	288	53.61	57	26	113	28	56	8	227	80	225	44	Yes
28	Wang, Longqing^a^	2001	Guangdong	264	52.27	65	37	75	38	39	10	205	112	153	58	Yes
29	Hu, Sheng^a,b,c^	2001	Hubei	168	55.36	49	30	48	24	16	1	146	84	80	26	Yes

HWE: Hardy-Weinbery equilibrium; a: The distribution of gender between case and control group is in balance; b: The distribution of age between case and control group is in balance; c: The distribution of BMI between case and control group is in balance.

### Results of the Overall Meta-Analysis

Table 2 showed the ORs with their 95% CIs for the association between MTHFR C677T polymorphism and T2DM in the recessive, dominant, homozygous, heterozygous, and additive genetic model. There was a significant association between MTHFR C677T polymorphism and T2DM under dominant (OR: 1.70, 95% CI: 1.42–2.02), recessive (OR: 1.48, 95% CI: 1.21–1.80), homozygous (OR: 1.89, 95% CI: 1.47–2.42), heterozygous (OR: 1.58, 95% CI: 1.33–1.87), and additive (OR: 1.46, 95% CI: 1.28–1.68) genetic model in a random-effects model.

**Table 2:** The overall and stratified analysis for the association between MTHFR and T2DM.

Genetic Model	Subgroup	Model for meta-analysis	OR(95% CI)	*P* for heterogeneity	I^2^ (%)	*P* for Egger's test
**Dominant**	overall	R	1.70(1.42–2.02)	0.00	56.9	0.45
	**Region**					
	Southern China	R	1.71(1.32,2.21)	0.04	49.6	
	Northern China	R	1.68(1.32,2.14)	0.00	61.9	
	**HWE**					
	Yes	R	1.73(1.39,2.15)	0.00	60.5	
	No	F	1.57(1.28,1.93)	0.07	47.8	
**Recessive**	overall	R	1.48(1.21–1.80)	0.02	37.7	0.00
	**Region**					
	Southern China	F	1.70(1.29–2.23)	0.81	0.00	
	Northern China	R	1.39(1.07–1.81)	0.01	50.4	
	**HWE**					
	Yes	R	1.61(1.23–2.09)	0.01	44.3	
	No	F	1.28(1.00–1.63)	0.34	11.6	
**Homozygous**	overall	R	1.89(1.47–2.42)	0.00	50.0	0.01
	**Region**					
	Southern China	F	2.07(1.56,2.76)	0.60	0.00	
	Northern China	R	1.81(1.28,2.56)	0.00	62.1	
	**HWE**					
	Yes	R	2.13(1.53,2.95)	0.00	53.2	
	No	F	1.51(1.16,1.96)	0.18	32.6	
**Heterozygous**	overall	R	1.58(1.33–1.87)	0.00	46.4	0.33
	**Region**					
	Southern China	R	1.57(1.18,2.08)	0.03	52.3	
	Northern China	R	1.58(1.28,1.97)	0.02	46.0	
	**HWE**					
	Yes	R	1.59(1.30,1.95)	0.00	51.1	
	No	F	1.52(1.20,1.92)	0.16	35.1	
**Additive**	overall	R	1.46(1.28–1.68)	0.00	64.5	0.01
	**Region**					
	Southern China	F	1.53(1.34,1.75)	0.29	16.6	
	Northern China	R	1.42(1.17,1.72)	0.00	72.7	
	**HWE**					
	Yes	R	1.48(1.26,1.75)	0.00	66.9	
	No	R	1.41(1.11,1.78)	0.02	64.5	

OR: odds ratio; R: random-effects model; F: fix-effects model. HWE: Hardy-Weinbery equilibrium

### Meta-Regression and Stratified Analysis

There was a significant heterogeneity in each genetic model (Table 2). we used meta-regression to explore the sources of heterogeneity in each genetic model separately. Similarly, heterogeneity can be explained by the number of the control group in each genetic model (Table 3).

**Table 3:** The results of meta-regression in the five genetic models.

Genetic Model	Variables	*P* for meta-regression
**Dominant**	year	0.521
	total number of study	0.175
	number of control	0.008
	number of case	0.504
	male (%)	0.152
**Recessive**	year	0.534
	total number of study	0.738
	number of control	0.013
	number of case	0.530
	male (%)	0.396
**Homozygous**	year	0.479
	total number of study	0.373
	number of control	0.003
	number of case	0.995
	male (%)	0.347
**Heterozygous**	year	0.580
	total number of study	0.150
	number of control	0.028
	number of case	0.367
	male (%)	0.152
**Additive**	year	0.683
	total number of study	0.419
	number of control	0.008
	number of case	0.952
	male (%)	0.116

In the subgroup analysis based on region, we divided the included studies into two major group, the northern and the southern [20]. The northern group included Beijing, Gansu, Heilongjiang, Hebei, Tianjin, Jilin, Neimenggu, Shandong, Shanxi, and the southern group included Hubei, Jiangsu, Shanghai, Guizhou, Zhejiang, and Guangdong. There was a significant association between MTHFR C677T polymorphism and T2DM under each genetic model in both groups. Likewise, we performed subgroup analysis on studies in which the MTHFR alleles in the control group were in HWE and on studies in which they were not in HWE, there was a significant association between MTHFR C677T polymorphism and T2DM under each genetic model in both groups (Table 2).

### Sensitivity Analysis

Table 4 showed the pooled ORs and their 95%CIs of sensitivity analysis by excluding one study at a time in each genetic model, the results in the five genetic models indicated that the overall result was dependable.

**Table 4:** Sensitivity analysis by removing each study in each model.

Study Removed	Dominant	Recessive	Homozygous	Heterozygous	additive
	OR(95% CI)	OR(95% CI)	OR(95% CI)	OR(95% CI)	OR(95% CI)
**Sun, Liang**	1.73(1.45,2.07)	1.49(1.31,1.82)	1.92(1.49,2.48)	1.61(1.36,1.91)	1.48(1.29,1.71)
**Mei, Qingbu**	1.74(1.47,2.07)	1.52(1.25,1.85)	1.97(1.54,2.51)	1.61(1.36,1.91)	1.49(1.31,1.71)
**Dai, Hongshuang**	1.71(1.42,2.04)	1.45(1.19,1.76)	1.86(1.45,2.39)	1.59(1.34,1.90)	1.46(1.27,1.68)
**Chen, Airong**	1.66(1.39,1.98)	1.44(1.18,1.76)	1.83(1.43,2.35)	1.55(1.31,1.84)	1.44(1.26,1.65)
**Zhang, Qiaohui**	1.67(1.40,2.00)	1.46(1.19,1.79)	1.86(1.44,2.40)	1.56(1.31,1.85)	1.45(1.26,1.66)
**Qiu, Yi**	1.70(1.42,2.04)	1.49(1.21,1.83)	1.91(1.47,2.48)	1.58(1.33,1.89)	1.47(1.27,1.69)
**Hu, Ling**	1.68(1.40,2.00)	1.46(1.19,1.78)	1.86(1.44,2.40)	1.56(1.31,1.86)	1.45(1.26,1.66)
**Wen, Jie**	1.68(1.40,2.01)	1.46(1.19,1.78)	1.85(1.44,2.38)	1.56(1.31,1.86)	1.45(1.26,1.67)
**Luo, Dan**	1.71(1.42,2.05)	1.50(1.22,1.84)	1.93(1.49,2.49)	1.58(1.33,1.89)	1.47(1.28,1.70)
**Chen, Ping**	1.74(1.47,2.07)	1.52(1.25,1.85)	1.97(1.55,2.50)	1.61(1.37,1.91)	1.50(1.31,1.71)
**Zhang, Chunyu**	1.69(1.41,2.02)	1.48(1.21,1.82)	1.90(1.46,2.45)	1.57(1.32,1.87)	1.46(1.27,1.68)
**Luo, Dan**	1.70(1.41,2.04)	1.51(1.24,1.85)	1.92(1.48,2.49)	1.57(1.31,1.87)	1.47(1.28,1.69)
**Yue, Hong**	1.66(1.39,1.97)	1.45(1.19,1.76)	1.83(1.43,2.35)	1.55(1.31,1.83)	1.44(1.26,1.65)
**Xiao, Yan**	1.63(1.38,1.91)	1.46(1.20,1.79)	1.85(1.45,2.37)	1.50(1.30,1.74)	1.43(1.25,1.63)
**Sun, Ying**	1.70(1.41,2.04)	1.45(1.19,1.78)	1.91(1.47,2.49)	1.59(1.33,1.89)	1.45(1.26,1.67)
**Shi, Chengjun**	1.72(1.44,2.06)	1.48(1.21,1.81)	1.91(1.48,2.47)	1.60(1.35,1.91)	1.47(1.28,1.70)
**Liang, Wenchang**	1.72(1.44,2.05)	1.48(1.21,1.81)	1.91(1.48,2.46)	1.60(1.35,1.90)	1.47(1.28,1.69)
**Guo, Lixin**	1.71(1.42,2.05)	1.51(1.22,1.85)	1.93(1.49,2.51)	1.58(1.33,1.89)	1.47(1.28,1.70)
**Sun, Jiazhong.**	1.70(1.42,2.05)	1.50(1.22,1.84)	1.92(1.48,2.50)	1.58(1.32,1.88)	1.47(1.27,1.69)
**Zhou, Jun**	1.72(1.44,2.05)	1.52(1.26,1.84)	1.95(1.52,2.50)	1.59(1.33,1.88)	1.49(1.31,1.71)
**Sun, Lei**	1.65(1.39,1.96)	1.42(1.18,1.72)	1.80(1.42,2.28)	1.54(1.30,1.83)	1.43(1.25,1.63)
**Mao, Li**	1.70(1.42,2.03)	1.47(1.20,1.79)	1.90(1.47,2.44)	1.58(1.33,1.88)	1.46(1.27,1.68)
**Chen, Airong**	1.65(1.39,1.97)	1.47(1.20,1.80)	1.85(1.44,2.38)	1.54(1.30,1.81)	1.44(1.26,1.65)
**Xu, Jinsheng**	1.74(1.47,2.07)	1.52(1.25,1.85)	1.97(1.55,2.50)	1.61(1.30,1.90)	1.50(1.31,1.71)
**Zhang, Guodong**	1.70(1.41,2.04)	1.47(1.20,1.81)	1.88(1.46,2.44)	1.58(1.32,1.88)	1.47(1.27,1.69)
**Shi, Jieping**	1.69(1.41,2.02)	1.48(1.21,1.81)	1.88(1.46,2.42)	1.57(1.32,1.86)	1.48(1.29,1.70)
**Yang, Guoqing**	1.68(1.40,2.01)	1.45(1.19,1.77)	1.85(1.44,2.39)	1.57(1.32,1.87)	1.45(1.26,1.67)
**Wang, Longqing**	1.71(1.43,2.05)	1.46(1.19,1.78)	1.88(1.46,2.44)	1.60(1.35,1.90)	1.47(1.27,1.69)
**Hu, Sheng**	1.70(1.42,2.04)	1.44(1.19,1.75)	1.85(1.45,2.36)	1.59(1.34,1.89)	1.46(1.27,1.67)

### Assessment of Publication Bias

As shown in table 2, Egger's test suggested no publication bias in dominant and heterozygous, but not in recessive, homozygous and additive genetic model. Because of this, we used the trim and fill method, the pooled analysis incorporating the hypothetical studies continued to show a statistically significant association between MTHFR C677T polymorphism and T2DM under recessive (OR: 1.26, 95% CI: 1.02–1.54), homozygous (OR: 1.60, 95% CI: 1.23–2.08) and additive (OR: 1.29, 95% CI: 1.12–1.49) genetic model.

## Discussion

This current study, to our knowledge, was the first to use a meta-analysis to evaluate the association between MTHFR C677T polymorphism and T2DM specifically in China. There was a significant relationship between MTHFR C677T polymorphism and T2DM in each genetic model. The prevalence of MTHFR C677T polymorphism varies in the different regions in China [20], so we separated northern group from southern group, and still got similar results, which compared to the overall results. According to whether HWE in control, we also found that there was a significant association between MTHFR C677T polymorphism and T2DM in each genetic model. Sensitivity analysis indicated there was no significant change on the overall results by removing one study in each turn. Egger's test suggested publication bias in recessive, homozygous and additive genetic model. The trim and fill analysis did not change the general results in the three genetic models (although the strength of the association was slightly attenuated), suggesting that the results of our analysis were credible. Based on the results of our meta-analysis, we can speculate that MTHFR 677T allele might increase the risk of T2DM in the Chinese Han population.

As an essential intermediate, homocysteine plays an important role between floate and activated methyl cycle, which is involved in the transfer of activated methyl groups from tetrahydrofolate to S-adenosylmethionine [52]. The methyl cycle has effects on global and gene promoter-specific DNA methylation in regulating gene expression [53], [54]. Some studies suggested that homocysteine exposure had adverse effects on beta cell glucose metabolism and cell viability, and impaired insulin secretory function [55]. There was a significant association between homocysteine level and insulin resistance [9], [56]. Due to its biological relevance and its association with metabolic disorders, homocysteine metabolism is an important candidate pathway for T2DM. The C677T variant of MTHFR plays an important role on homocysteine metabolism [57]. The homozygous 677TT and heterozygous 677CT genotypes have decreased 70% and 35% in the enzyme activity of MTHFR respectively, compared to the 677CC genotype [58]. Individuals with the homozygous 677TT genotype have higher plasma homocysteine and lower plasma folate levels than those with 677CC genotype [59]. MTHFR C677T polymorphism has also been reported to be associated with type 2 diabetes, and its complications [17], [30], .

The variation of MTHFR A1298C polymorphism, which was an A to C transition at base pair 1298 resulting in the amino acid transition from Glu to Ala, could also decrease enzyme activity, and lead to hyperhomocysteinemia. The A1298C variation was located in the C-terminal regulatory domain of the MTHFR gene, while the C677T variation was located in the gene catalytic domain [61]. The A1298C variation had lower impact on the enzyme activity, compared with the C677T variation [61]. So the majority of studies on T2DM mainly paid attention to the C677T variation, not the A1298C variation, and we only collected one study on the relationship between MTHFR A1298C polymorphism and T2DM in Chinese Han population before December 2013 [37]. Therefore, in our meta-analysis, we only considered the studies on the relationship between MTHFR C677T polymorphism and T2DM.

In 2013, Khalid et al. found that there was a significant association between MTHFR C677T polymorphism and T2DM in Arab population [14], and Zhong et al. also conducted a meta-analysis of the relationship between MTHFR C677T polymorphism and T2DM, and concluded that there was no association between MTHFR C677T polymorphism and T2DM, regardless of the ethnicity of the patient or the presence of serious DM-related complications [13]. Our meta-analysis showed a significant relationship between MTHFR C677T polymorphism and T2DM under five genetic models in Chinese Han population. The results in our meta-analysis were similar to Khalid's study, and different from Zhong's study. There are several reasons for this difference. First, Zhong et al. conducted the meta-analysis all over the world, only loosely classified the study population as African, Asian, or Caucasian. Because MTHFR C677T polymorphism distribution varies among different ethnic groups, the relationship between MTHFR C677T polymorphism and T2DM should be studied on a single ethnic group. Therefore, our study focused on the Chinese Han population to derive an accurate evaluation. Second, more than a third of included studies focused on the Chinese Han population in Zhong's study, but he just conducted subgroup analysis in Asian population, did not further analyze the association in Chinese Han population. Third, Zhong's study only included 16 studies on the Chinese Han population, while our study included 29 studies. We think the number of included studies for Zhong' meta-analysis was inadequate, for example Sun et al. [15], Qiu et al. [28], Chen et al. [32] and so on. The findings suggested that we need to further analyze the association between MTHFR C677T polymorphism and T2DM in the Chinese Han population.

There are several limitations in our meta-analysis. First of all, the subjects in the included studies were too small, so more large-scale studies were needed to assess the association between MTHFR C677T polymorphism and T2DM. And due to lack of necessary personal information in the included studies, we were unable to further perform subgroup analysis for the relevant influential factors (gender, age, BMI and so on). Second, all included studies were cross-sectional design and all the subjects came from hospitals, their results were not adjusted by the relevant influential factors. They could not infer cause-effect relationship. Third, the development of T2DM was affected by the multiple genes, and our meta-analysis only focuses on MTHFR C677T polymorphism, so the influence of MTHFR C677T polymorphism on T2DM may be affected with other gene polymorphism. Some studies had suggested that ACE insertion/deletion (I/D) polymorphism may act synergistically with MTHFR C677T polymorphism to increase the risk of T2DM [62].

In conclusion, our meta-analysis suggested there was a significant association between MTHFR C677T polymorphism and T2DM in the Chinese Han population, and indicated that MTHFR 677T allele might be a risk genetic factor in developing T2DM. Because of the limitations in our study, more large-scale studies need consider the relevant influential factors and other gene polymorphism to verify the results of our study.

## Supporting Information

Checklist S1 **PRISMA Checklist of this systematic review.**
(DOC)

Checklist S2 **Meta-analysis on Genetic Association Studies Checklist.**
(DOCX)
